# Network Dynamics of Social Influence on E-cigarette Use Among an Ethnically Diverse Adolescent Cohort

**DOI:** 10.1093/ntr/ntaf065

**Published:** 2025-03-16

**Authors:** Sarah E Piombo, Kayla de la Haye, Thomas W Valente

**Affiliations:** Department of Population and Public Health Sciences, Keck School of Medicine, University of Southern California, Los Angeles, CA, USA; Center for Economic and Social Research, Dornsife College of Letters, Arts and Sciences, University of Southern California, Los Angeles, CA, USA; Department of Population and Public Health Sciences, Keck School of Medicine, University of Southern California, Los Angeles, CA, USA

## Abstract

**Introduction:**

The objective of this study was to examine the mechanisms of social influence driving e-cigarette use in adolescent social networks and differentiate between the effects of exposure to friend behavior and social norms on individual use.

**Aims and Methods:**

Surveys on health behaviors and friendship networks from nine high schools in Southern California (*N* = 2245; 48% Hispanic) were collected at three time points from Spring 2021 of grade 9, Fall 2021, and Spring 2022 of grade 10. Stochastic actor-oriented models for the co-evolution of social networks and behavior dynamics tested for friendship network social influences on e-cigarette use. Two mechanisms of social influence were estimated, exposure to friend behavior (e-cigarette use among friends) and pro-e-cigarette social norms (perceived peer approval and use of e-cigarettes), while controlling for social selection, individual covariates, and endogenous network effects. Results from the nine schools were combined in a meta-analysis.

**Results:**

Findings revealed social influence effects through exposure to friend e-cigarette use and pro-e-cigarette social norms, which both had significant positive influences on individual e-cigarette initiation over time. Furthermore, Hispanic/Latine individuals and females were more likely to initiate e-cigarette use compared to males and non-Hispanic/Latine students.

**Conclusions:**

The importance of these effects should be considered in tobacco prevention initiatives. Designing culturally tailored interventions that target youth social networks and e-cigarette social norms could be effective at curtailing adolescent use. Changing perceptions and social acceptability of e-cigarettes could be one way to slow or prevent the spread of e-cigarette use in adolescent networks.

**Implications:**

These findings demonstrate the role of social influence on adolescent e-cigarette use through two mechanisms, both friend behavior and social norms. A shift in prevention strategies toward addressing social dynamics and social norms rather than focusing solely on individual-level factors may be effective in curtailing use. Changing social perceptions and reducing the social acceptability of e-cigarette use may help slow the spread in adolescent networks.

## Introduction

E-cigarettes have been the most popular tobacco product among adolescents for the last 10 years.^1,2^ In 2024, 7.8% of high school adolescents in the United States reported past 30-day e-cigarette use.^3^ Alaska Native/American Indian, Hispanic/Latine, and non-Hispanic multi-racial students had the highest rates of e-cigarette use compared to other racial or ethnic groups.^3^ While e-cigarette use declined among adolescents between 2023 and 2024, the introduction of e-cigarettes has begun a new era for the tobacco industry, providing opportunities to target younger audiences and reignite youth consumption. Clever marketing and social media campaigns have distanced e-cigarettes from the social and health risk stigmas of combustible cigarettes in the minds of many adolescents and adults.^4–7^ However, the Centers for Disease Control and Prevention and surgeon general have declared that e-cigarettes are not safe for youth and have issued a call for increased tobacco control strategies to reduce and prevent e-cigarette use.^3^ The popularity of e-cigarettes now echoes the public health battle with combustible cigarettes among youth in generations past: addictive, popular, and difficult to regulate.

The popularity, acceptability, and heightened public salience of e-cigarette use lead to distinct social dynamics that can accelerate e-cigarette uptake, particularly among adolescents. Generally, when people are exposed to e-cigarettes in both real-life social networks and on social media, they are more likely to adopt this behavior as a result of social influence.^7^ Adolescents are in a development stage that is often characterized by the desire to fit in with friends and be socially accepted by peers,^8^ which heightens their awareness of social norms and susceptibility to peer influence. Since adolescent e-cigarette use is likely to be socially influenced, social network analysis (SNA) is a valuable tool to understand that influence and yield insights about the social spread of e-cigarette use and potential intervention strategies to curtail use.

### Social Network Framework

SNA is a defined theoretical perspective and set of methods used to analyze and understand relationships between people and how they affect individual beliefs and behaviors, and broader group outcomes.^9^ Social relationships and interactions expose people to others’ behaviors and the exchange of information, opinions, role modeling, and normative influences. SNA methods provide useful tools to study social influence in the context of adolescent behavior and to model network and behavior dynamics jointly and thus parse out processes of social selection and social influence.^10^ Although many adolescents’ social networks are comprised of relations to family, friends, and other contacts, SNA research has shown that their dynamic relationships with same-age peers are a social context with a powerful influence on their health and risk-taking behaviors.^11^

In studies of adolescent social networks and smoking, there is strong evidence that peer smoking is positively associated with individual smoking.^10,12–28^ Longitudinal studies of adolescent smoking and social networks, that model mechanisms driving these relationships over time, have further established that social selection and influence are important. There is consistent evidence of peer selection effects, where individuals are likely to form friendships with others who have similar smoking behaviors.^17,18,23,26,28,29^ However, adolescents are unlikely to begin smoking without being exposed to cigarette use among friends first. In adolescent networks, the likelihood of being a current smoker increases when smoking is prevalent in personal networks or among one’s best friends.^20,26,30^ Together, these findings demonstrate the importance of peer influence and network exposure on adolescent smoking even while accounting for endogenous factors that predict network change. While the relationship between peer influence and smoking is established, there are limited studies exploring the same social phenomena and mechanisms driving e-cigarette use among youth. One study found that e-cigarette use among friends was associated with individual e-cigarette initiation and use.^31^ However, more longitudinal studies are needed to examine these trends and other factors associated with use. The greater prevalence and popularity of e-cigarettes compared to combustible cigarettes suggests that there are distinct differences in their social acceptability and social dynamics driving use. The social visibility of e-cigarette use dispels notions of harm and normalizes the behavior. Given these differences and the popularity of e-cigarettes among adolescents over the last decade, it is important to understand the mechanisms of peer influence driving uptake in order to effectively address it.

### Peer Influence and Perceived Norms

Understanding the mechanisms through which peer influence occurs is critical to designing effective interventions that target the appropriate mechanism. Two key mechanisms that play a role in peer influence among youth are social influence through direct behavior exposure and social influence through behavioral norms. For e-cigarette use, peer e-cigarette exposure can be quantified as the proportion of friends in one’s social network that self-report using e-cigarettes.

Social norms are *people’s beliefs* about what is typical. They can be defined as descriptive norms, perceptions about others’ behavior, or injunctive norms, perceptions of approval or acceptable behavior.^32,33^ For e-cigarette use, the role of perceived norms, such as one’s perception of e-cigarette use prevalence among their peers, and perceptions of whether or not peers approve of e-cigarettes, could be mechanisms of social influence. The positive association between perceptions of peer smoking and individual smoking has been strongly demonstrated among Hispanic/Latine adolescents.^21^ However, adolescents may inaccurately overestimate how prevalent e-cigarette use is among friends due to the increased visibility of the behavior, as modeled by their friends, popular peers, or the media, and thus have inaccurate perceived norms. Social networks are instrumental in informing and communicating social norms and can be leveraged to correct incorrect views about behavior prevalence and acceptability.^32^ One objective of this study is to differentiate between these mechanisms of social influence—through direct peer exposure and behavioral norms—to determine if either or both mechanisms influence e-cigarette use in adolescent social networks.

### Examining the Co-evolution of Friendship Networks and E-cigarette Use

This study addresses a gap in our current understanding of how adolescent social networks are linked to e-cigarette use. We focus on a cohort of 2245 youth across nine high schools from different socio-economic settings in Southern California. Our study population is 48% Hispanic/Latine, the ethnic group with the highest rate of e-cigarette use among adolescents.^2^ The study models peer social networks and e-cigarette behavior dynamics in a diverse adolescent cohort, to test for social influence on e-cigarette use. Because no studies, to the best of our knowledge, have explored specific mechanisms of social influence on e-cigarette use, a key aim is to determine if youth e-cigarette use is influenced by direct network exposure, perceived pro-e-cigarette norms, or both.

## Materials and Methods

### Participants and Recruitment

Assessing Developmental Patterns of Vaping, Alcohol, Nicotine, and Cannabis Use and Emotional Well-being (ADVANCE) is a prospective cohort study on the health behaviors of high school students in the Southern California area.^34^ Data collection began in August 2020 with a pilot subset and then continued in January 2021 with the full sample. Students were surveyed each Fall and Spring semester. This analysis uses the first three full waves of data from January 2021, August 2021, and January 2022 across nine high schools, with each high school considered one social network. At wave 1 (Spring/January 2021), across eleven schools 3968 eligible students were invited to participate. Of these, 2211 students provided parental consent, student assent, and completed the survey. One school did not participate in the SNA portion of the survey, and one school’s model ultimately did not converge so these schools were removed from the final analysis. The analytic sample was restricted to individuals who participated in at least two waves of data collection without missing data on the dependent behavior variable (e-cigarette use; *N* = 1510 in Spring 2021). Additional participants joined the study at subsequent waves resulting in an analytic sample of 2245 students across nine schools/networks and three waves of data collection. Among the nine high schools, three are in low-income, low-resource settings, two are in middle to high-income settings, and the remaining four schools are in middle-income urban settings. The schools all comprise racially and ethnically diverse student populations. Participants were recruited in 9^th^ grade (ages 13–15) at baseline. Study approval was granted by the (removed for anonymized review) IRB.

### Measures

#### Friendship Networks

Friendship network data was collected through a survey question that stated: “Name up to seven (7) of your closest friends in your grade at school in the spaces below. Enter your friends first and last real name, not their nickname.” School rosters were pre-loaded so that names would populate as students began typing. This nomination method has been successfully deployed in several studies, including school-based adolescent studies and provides valid and reliable network data.^9,19,21,31^ Friendship nominations were used to construct social networks for each school, at each study wave.

### E-cigarette Use and E-cigarette Norms

The outcome was “ever used e-cigarettes,” defined as a dichotomous behavioral outcome *(“never used” = 0 vs. “ever used” = 1*). Perceived pro-e-cigarette norms were measured with items that reflected descriptive norms (perceptions about network e-cigarette use) and injunctive norms (peer approval of e-cigarette use) within one’s social network. The following questions were asked: *“*People who are important to me use e-cigarettes; My friends don’t mind when other people use e-cigarettes around them; E-cigarettes are more socially acceptable than smoking cigarettes; A lot of people vape e-cigarettes*” (Response options 0 = disagree; 1 = Don’t know; 2 = Agree)*. Confirmatory factor analysis was used to assess construct validity (see Appendix A Table S1 for details) and an average summary score of perceived pro-e-cigarette norms was calculated, with higher scores indicating greater perceived pro-e-cigarette norms. We controlled for demographic covariates sex at birth (*female = 0, male = 1*) and ethnicity (*non-Hispanic = 0, Hispanic = 1*).

## Analysis

Stochastic actor-oriented models (SAOM) are agent-based models for the co-evolution of social networks and behavior change.^35,36^ SAOM models the changes in a social network from the perspective of the individuals (ie, “actors”). The model is a continuous-time Markov chain, where changes occur through a series of unobserved mini-steps, in which actors have the option of forming or dissolving friendships or changing their behavior. These longitudinal models estimate the effect of factors that might influence these decisions, such as the network dynamics and the characteristics of the actors, which are specified as parameters in the model.^37^ SAOM have been used to examine the co-evolution of network changes and health behaviors or outcomes such as physical activity,^38^ tobacco use,^18,25^ marijuana use,^39^ and sexually transmitted infections.^40^

SAOM have two components or processes: (i) network evolution, where friendship changes (network tie changes) among actors is the dependent variable, and (ii) behavior evolution, where behavior changes among actors is the dependent variable. The co-evolution of the network and behavior is estimated using the method of moments implemented by computer simulation of the change processes.^36^ Factors that influence changes in the friendship network are called selection effects, while factors that influence changes in behavior are called influence effects. Since the co-evolution of the network and behavior are measured by distinct processes, SAOM are a useful analytic technique for differentiating the effects of social selection from social influence, which may both give rise to correlations between a behavior and social connections.^10,18,19,23,24,26,28,29^

SAOM were estimated for each of the nine high school networks separately using the RSiena package.^37^ Modeling the e-cigarette behavior variable as initiation/ever-use allows us to estimate the rate at which e-cigarette use spreads throughout the network and parameters that impact behavior diffusion.^41^ Details of SAOM estimation and the RSiena package are described elsewhere.^10,36^ We used these models to test the effects of social influence on adolescent e-cigarette use over time, controlling for social selection and other confounding effects.

### Co-variate Effects for Friend Selection

In these models, the network evolution part estimated selection effects that predict the formation, maintenance, or dissolution of friendships. Selection effects were chosen based on model requirements and previous research. We included three types of effects that represent how individual attributes affect selection: (i) *ego effects* are the tendency for individuals with a given characteristic to send friend nominations, (ii) *alter effects* are the tendency for individuals with a given characteristic to receive friend nominations, and (iii) *same (or similarity) effects* are the tendency for individuals to form friendships with others who are the same/similar to themselves on a given characteristic. For example, using sex as an attribute, a positive f*emale ego* effect would indicate that females are more likely to send friendship nominations, a positive *female alter* effect would indicate that females are more likely to receive friendship nominations, and a positive *female homophily* effect would indicate that individuals are more likely to form a friendship with others of the same sex. These three effects, or terms, represent network evolution processes based on individual attributes and we included them for sex and ethnicity. Ego, alter, and homophily effects for e-cigarette use were also initially included in order to estimate changes in the friendship network based on the behavior of interest. As we detail in the results these e-cigarette selection terms could not be estimated.

### Endogenous Network Effects for Friend Selection

The model also included effects that represent endogenous network processes known to contribute to the formation of friendships. Degree-related effects measure the balance of friendship ties made and received. The in-degree popularity effect measures the tendency for popular individuals to continue receiving more friendship nominations, while the out-degree popularity effect measures the tendency for popular individuals to continue sending more nominations. The in-degree activity effect measures the tendency of individuals who send many friendship nominations to receive more friendship nominations.

Reciprocity measures the tendency for friendship nominations to be reciprocated or mutual. The geometrically weighted edgewise shared partnerships (GWESP) effect measures triadic closure, or the tendency for individuals to form ties with friends of friends. The model assumes that individuals have the opportunity to form or dissolve friendships at each mini-step in the MCMC simulation, modeled by the rate of these changes for each period between observed data points.

### Social Influence Effects on E-cigarette Use

The behavior evolution part of the model includes effects that predict changes in e-cigarette use over time based on individual and network characteristics. Our primary aim is to test for social influence and determine if it is driven by direct exposure to peer e-cigarette use among networks and/or perceived pro-e-cigarette norms. Social influence was operationalized by two effects: (1) the effect of friend e-cigarette use (peer exposure), and (2) perceived pro-e-cigarette norms, in predicting adolescent e-cigarette use. The results will provide insight into how e-cigarette initiation and use spreads through adolescent peer networks over time.

Exposure to peer e-cigarette use is tested by the average exposure effect on the rate of e-cigarette use behavior. Peer exposure is calculated based on the proportion of one’s friends that self-report e-cigarette use for that time period. For example, if an individual has five friends and four of those friends report using e-cigarettes, then peer exposure to e-cigarette use would be 80% at that time point. The average exposure effect measures whether increases in exposure are associated with an increased rate of e-cigarette use.

Perceived pro-e-cigarette norms are tested by the pro-e-cigarette norms’ effect on the rate of e-cigarette use behavior (composite measure described above). The model also controls for other individual factors that could be predictive of behavior change, such as sex and ethnicity.

### SAOM Building

A preliminary model was fit for two schools using forward model selection with score-tests (t-ratios, estimate divided by standard error) to test the significance of parameters to predict co-evolution of friendship networks and e-cigarette use initiation. The same model was then fit to all nine schools. To obtain a parsimonious model that could be fit across all networks we chose effects that were theoretically relevant to our research question and/or important to control for based on prior research. If a parameter could not be estimated for a specific network, the effect was set to zero for that network to retain the network in the meta-analysis which requires the same model specification across networks. Overall convergence ratios for each of the nine networks were <0.25 with convergence t-ratios for all parameters <0.10.

### Siena Meta-analysis

A two-step meta-analytic approach was used to combine results from the nine networks.^37,42^ Each high school grade network is considered an independent group sampled from a population (of dynamic networks) and results are combined for each parameter. The dependent variable is the population parameter estimate which is the result of combining the parameter estimates from each individual network. All analyses were implemented using RSiena (Simulation Investigation for Empirical Network Analysis)**^37^** for the statistical system R version 4.3.^43^

## Results

**Figure 1.**
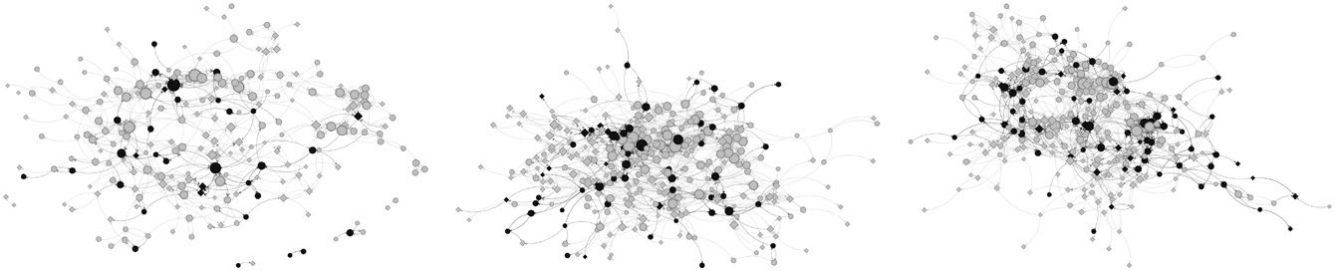
A high school network at three-time points: Spring 2021 (left panel), Fall 2021 (middle panel), and Spring 2022 (right panel). Dark nodes indicate e-cigarette users. Circular nodes are female, square nodes are male. Nodes are sized by in-degree (number of friendship nominations received).

Descriptive statistics for demographic and network characteristics of the analytic sample are presented in Tables 1 and 2. Almost half of the participants were Hispanic/Latine ethnicity which is typical for Southern California. At baseline, the average network size was 167.8 students, with a 5.4% prevalence of e-cigarette use (See Appendix A Table S2 for separate school network metrics). Regressions were run to determine if any characteristics were associated with being lost to follow-up (not shown). No covariates were associated with participants lost to follow up from wave 1 to wave 2, however, Hispanic/Latine students were more likely to be lost to follow up from wave 2 to wave 3. Figure 1 shows one of the high school friendship networks at each time point with nodes colored to show e-cigarette use status. The results of the SAOM meta-analysis are described below, focusing on predictors of friendship network dynamics first, and then predictors of e-cigarette use, including the two social influence effects of interest.

**Table 1.** Demographic and Behavior Characteristics (*N* = 2245)

	*N* (%)
Sex assigned at birth	
Male	1006 (44.8)
Female	1225 (54.6)
Missing	14 (0.6)
Ethnicity	
Hispanic	1079 (48.1)
Missing	2 (0.1)
Race	
American Indian/Alaska Native	46 (2.0)
Asian	734 (32.7)
Black or African American	44 (2.0)
Native Hawaiian/Pacific Islander	12 (0.5)
White	361 (16.0)
Multi-racial	511 (22.8)
Other	467 (20.8)
Missing	70 (3.1)
E-cigarette user	
Wave 1	122 (5.4)
Wave 2	228 (10.2)
Wave 3	287 (12.8)
**Mean (SD)**	
Age (years)	
Wave 1	14.64 (0.6)
Wave 2	15.51 (0.4)
Wave 3	15.96 (0.4)
Pro-e-cigarette norms^	
Wave 1	3.81 (1.8)
Wave 2	3.91 (2.0)
Wave 3	—

^ range 0–8; norms not measured at wave 3.

**Table 2.** Social Network Characteristics (*N* = 9 Networks)

Average network metrics	Wave 1	Wave 2	Wave 3
Size (number of individuals)	167.8 (81.1)	210.1 (93.5)	214.1 (84.9)
Edges (friendship ties)	670.9 (620.8)	611.2 (389.3)	620.9 (366.9)
Reciprocity (proportion)	0.52 (0.06)	0.50 (0.07)	0.51 (0.05)
Density	0.02 (0.01)	0.01 (0.004)	0.01 (0.003)
Transitivity	0.29 (0.05)	0.26 (0.04)	0.26 (0.04)
Average path length	5.68 (1.7)	6.14 (1.3)	6.02 (1.05)

### Friendship Network Dynamics

The meta-analysis findings for changes in friendship networks and e-cigarette use are shown in Table 3. Additional meta-analysis details can be found in Table S3 in Appendix A. If an effect was unable to be estimated, or if the standard error exceeded an acceptable value, that network was dropped from the meta-analysis for that effect. Across networks, friendship ties were significantly more likely to be reciprocated, transitive, and with individuals of the same sex and ethnicity.

**Table 3.** Meta-analysis of Changes in Friendship Networks and E-cigarette Use in Nine High Schools (*N* = 2245)

Effect	School *N*	Estimate (s.e.)
*Network dynamics*		
Friend rate (period 1)	7	16.71 (1.39)***
Friend rate (period 2)	9	6.7 (0.66)***
Out-degree (density)	9	−3.41 (0.13)***
Reciprocity	9	2.90 (0.05)***
GWESP	9	1.73 (0.04)***
Popularity (in-degree square root)	9	0.19 (0.04)***
Popularity (out-degree square root)	9	−0.35 (0.04)***
Activity (in-degree square root)	9	−0.13 (0.07)***
Male		
Alter	9	0.04 (0.02)
Ego	9	0.06 (0.03)**
Homophily	9	0.50 (0.03)***
Hispanic		
Alter	9	0.03 (0.03)**
Ego	9	0.07 (0.03)**
Homophily	9	0.35 (0.06)***
Behavior dynamics		
Rate (period 1)	9	0.03 (0.01)
Rate (period 2)	9	0.02 (0.01)
Rate effects		
Average peer exposure	6	3.15 (0.96)*
Male	8	−0.66 (0.23)**
Hispanic	8	0.76 (0.22)**
Pro-e-cigarette norms	9	0.29 (0.06)***

*p < .05; **p < .01; ***p < .001.

*N* = number of schools on which the statistics for this effect were based;.

Estimate (${\hat{\mu }}_{\theta }$) = estimated average effect size; standard error (s.e.).

E-cigarette ego, alter, and same effects were initially estimated but omitted from final models due to non-convergence.

GWESP = geometrically weighted edgewise shared partnerships

The reciprocity effect was significant and positive (${\hat{\mu }}_{\theta }$ = 2.90, *p* < .001), indicating that on average students tend to reciprocate friendship ties. Network transitivity was measured by the significant GWESP effect (${\hat{\mu }}_{\theta }$ = 1.73, *p* < .001), where there was a tendency to form ties with friends of friends. The positive in-degree popularity effect suggests that individuals with more friendship nominations tend to continue receiving more friendship nominations over time, increasing in popularity (${\hat{\mu }}_{\theta }$ = 0.19, *p* < .001). The negative out-degree popularity effects can be interpreted as individuals who send more friendship nominations are receiving fewer nominations over time (${\hat{\mu }}_{\theta }$ = −0.35, *p* < .001). The negative in-degree activity effect also suggests that more popular individuals tend to send fewer nominations over time (${\hat{\mu }}_{\theta }$ = −0.13, *p* < .001). Together, these can be interpreted as more popular students receiving, but not sending more friendship nominations. Additionally, there was evidence of demographic homophily, where individuals were more likely to form friendships with others of the same ethnicity and sex (${\hat{\mu }}_{\theta }$ = 0.50, *p* < .001; ${\hat{\mu }}_{\theta }$ = 0.35, *p* < .001). E-cigarette use selection effects were unable to be estimated and resulted in non-convergence. Please see Table S4 Appendix A for regression analyses examining friendship selection based on e-cigarette use.

### E-cigarette Use Behavior Dynamics

On average, the rate of e-cigarette use initiation had a positive, but non-significant, trend over each time period. Hispanic individuals had a significantly greater rate of e-cigarette use initiation compared to non-Hispanic students, while males had significantly lower e-cigarette use rates compared to females (${\hat{\mu }}_{\theta }$ = 0.76, *p* < .0’; ${\hat{\mu }}_{\theta }$ = −0.66, *p* < .01). While there was a significant correlation between peer exposure and pro-e-cigarette norms at wave 1 (*r* = .15, *p* < .001) and wave 2 (*r* = .16, *p* < .001), both of the hypothesized effects of social influence were supported. The average exposure effect of peer e-cigarette use on individual e-cigarette use was able to be estimated in six networks and was positive and significant in the meta-analysis (${\hat{\mu }}_{\theta }$ = 3.15, *p* < .05). Additionally, stronger pro-e-cigarette norms were significantly associated with an increased rate of e-cigarette use (${\hat{\mu }}_{\theta }$ = 0.29, *p* < .001). Thus, both mechanisms had independent and significant effects on adolescent e-cigarette use.

## Discussion

Peer influence plays a significant role in adolescent e-cigarette use. We explored peer influence effects through network exposure to peer e-cigarette use and perceived e-cigarette norms on individual e-cigarette use over time. To the best of our knowledge, this is the first study of its kind to explore these associations longitudinally among a large, ethnically diverse, adolescent cohort. Building on past social network and tobacco research,^18^ we used rigorous methodology to examine social influence factors associated with e-cigarette use while controlling for individual attributes and endogenous network effects. We found that peer influence operates through both network exposure to friends who vape and through perceived positive norms surrounding e-cigarette use.

On average, in our study sample e-cigarette use gradually increased over time along with peer exposure and pro-e-cigarette norms, demonstrating the increased tendency for engaging in risky behaviors during adolescence. As illustrated by one of the school networks in Figure 1, we can observe that changes in friendship networks and behaviors are dynamic and interrelated processes. As high school progresses, peer influence becomes more significant, with exposure to peers who use e-cigarettes or other substances further normalizing their use. As a result, the growing pro-e-cigarette social norms coupled with greater exertion of peer influence may make adolescents more likely to adopt e-cigarette use if it aligns with their social circles. Below, we further discuss these mechanisms of social influence.

These results provide evidence that peer influence has a persistent effect on adolescent e-cigarette use across different school contexts, and that having a higher proportion of friends who use e-cigarettes, and further, having pro-e-cigarette norms, increased the likelihood of e-cigarette use for these adolescents. The importance of these two effects could vary across contexts. For example, in schools with lower e-cigarette prevalence, students will have less opportunity for direct peer exposure, but overall positive attitudes towards e-cigarettes may exist nonetheless and encourage experimentation. The e-cigarette use prevalence in the schools in our study is relatively low at baseline (approximately 5%), which demonstrates that peer influence can still operate in a low-prevalence setting, and that the role of perceived pro-e-cigarette norms may become even more impactful in these networks.

While social network dynamics are nuanced and context-dependent, these findings show that addressing perceived norms and attitudes surrounding e-cigarette use could be a potential intervention strategy. Pro-e-cigarette norms are associated with e-cigarette use while controlling for peer exposure. While having friends that use e-cigarettes in one’s network has a significant effect on personal e-cigarette use, it can be difficult to create behavior change through social network alteration, which would entail discouraging or dissolving friendships with e-cigarette users. A norms messaging intervention aimed at reducing the social acceptability of e-cigarettes is potentially a more feasible and broadly applicable approach. In addressing social network norms, it will also become important to understand where pro-e-cigarette norms are originating, whether this is from social media, substance use among popular peers, or settings outside of school. Our study found that Hispanic/Latine students were at greater risk of initiating use, indicating that culturally tailored interventions and messaging strategies should also be considered.

### Strengths

This study has several strengths and some limitations. Estimating SAOM in RSiena allows us to model the changes in friendship networks and e-cigarette use behavior while controlling for non-independence of observations. Prior research has used these models with regard to cigarette smoking, but none to the best of our knowledge have examined e-cigarette use in a large, ethnically diverse, adolescent cohort. Additionally, Hispanic/Latine adolescents have the highest rate of e-cigarette use nationally, which highlights the need for increased understanding of e-cigarette use among this population. Another strength of our study is the meta-analysis, which allows us to make inferences about the underlying population that our networks are sampled from, giving our findings more generalizability compared to studies focusing on a singular network.

### Limitations

Inherent to longitudinal survey-based research, limitations of this study include missing data due to attrition or participants being lost to follow-up. Given the unique dynamics of networks, there was difficulty with fitting some of the effects across all networks. The low prevalence of e-cigarette use resulted in the average exposure effect being inestimable in three of the networks. Additionally, the low prevalence of e-cigarette use did not permit us to estimate the ego, alter, and similarity effects of e-cigarette use on friend selection, therefore we cannot draw any conclusions about individuals selecting friends based on e-cigarette use status.

## Conclusions

Our study is the first of its kind to explore friendship and e-cigarette use dynamics in a large, diverse, adolescent cohort. We built on past research by parsing social influence into peer exposure and normative effects and found that both factors exert influence on individual e-cigarette use behavior. Targeting perceived pro-e-cigarette norms may be an important strategy for creating behavior change, especially in networks with a low prevalence of e-cigarette use. Norms messaging interventions aimed at reducing positive attitudes and social acceptability of e-cigarettes may be instrumental in reducing the initiation and spread of e-cigarette use in adolescent networks. Social factors have a strong influence on adolescent health behavior, particularly risk behavior, and thus effective public health strategies require social responses.

## Supplementary Material

ntaf065_suppl_Supplementary_Materials

## Data Availability

The data underlying this article cannot be shared publicly because it contains the sensitive health information of minors. The data will be shared on reasonable request to the corresponding author.
