# Supplementary material for: Network Dynamics of Social Influence on E-cigarette Use Among an Ethnically Diverse Adolescent Cohort
Source: Nicotine Tob Res. 2025 Mar 16;27(9):1583–90. doi: 10.1093/ntr/ntaf065 (PMC12370464; doi:10.1093/ntr/ntaf065)
Supplement: ntaf065_suppl_Supplementary_Materials [file ntaf065_suppl_supplementary_materials.docx]

Appendix A

| **Supplemental Table 1. Perceived Pro-e-cigarette Norms Wave 1 and Wave 2 Confirmatory Factor Analysis** | | |
| --- | --- | --- |
| **Standardized Factor Loadings** | | |
|  | **Wave 1** | **Wave 2** |
| **Item** |  |  |
| *People who are important to me use e-cigarettes* | 0.57 | 0.66 |
| *My friends don’t mind when other people use e-cigarettes around them* | 0.48 | 0.55 |
| *E-cigarettes are more socially acceptable than smoking cigarettes* | 0.64 | 0.64 |
| *A lot of people vape e-cigarettes* | 0.67 | 0.73 |
| Cronbach's alpha | 0.58 | 0.64 |
| **Fit Indices** |  |  |
| CFI | 0.98 | 0.97 |
| TLI | 0.95 | 0.92 |
| RMSEA | 0.078 | 0.12 |
| SRMR | 0.040 | 0.057 |
| ^Standardized by the standard deviation of the factor and the item CFI = Comparative fit index; TLI = Tucker-Lewis index; RMSEA = Root mean square error of approximation; SRMR = Standardized root mean squared residual. | | |

| **Supplemental Table 2. School Friendship Network Characteristics** | | | | | |  |
| --- | --- | --- | --- | --- | --- | --- |
|  | **Wave 1** | | | | | |
| School | Size | Edges | Density | Reciprocity | Transitivity | APL |
| 101 | 169 | 429 | 0.02 | 0.59 | 0.38 | 6.42 |
| 102 | 58 | 80 | 0.02 | 0.48 | 0.23 | 3.91 |
| 103 | 131 | 228 | 0.01 | 0.53 | 0.25 | 7.63 |
| 104 | 222 | 597 | 0.01 | 0.54 | 0.29 | 7.39 |
| 105 | 80 | 134 | 0.02 | 0.54 | 0.27 | 3.84 |
| 106 | 93 | 256 | 0.03 | 0.50 | 0.32 | 2.91 |
| 112 | 255 | 1616 | 0.02 | 0.57 | 0.29 | 6.10 |
| 113 | 279 | 1666 | 0.02 | 0.54 | 0.32 | 6.38 |
| 114 | 223 | 1032 | 0.02 | 0.38 | 0.23 | 6.51 |
|  | **Wave 2** | | | | | |
| School | Size | Edges | Density | Reciprocity | Transitivity | APL |
| 101 | 212 | 595 | 0.01 | 0.55 | 0.25 | 6.14 |
| 102 | 113 | 169 | 0.01 | 0.35 | 0.24 | 2.84 |
| 103 | 117 | 252 | 0.02 | 0.56 | 0.26 | 6.78 |
| 104 | 282 | 870 | 0.01 | 0.51 | 0.28 | 6.75 |
| 105 | 101 | 240 | 0.02 | 0.52 | 0.29 | 6.51 |
| 106 | 140 | 269 | 0.01 | 0.41 | 0.22 | 6.85 |
| 112 | 299 | 1045 | 0.01 | 0.53 | 0.30 | 5.95 |
| 113 | 331 | 1144 | 0.01 | 0.54 | 0.31 | 7.08 |
| 114 | 296 | 917 | 0.01 | 0.48 | 0.21 | 6.31 |
|  | **Wave 3** | | | | | |
| School | Size | Edges | Density | Reciprocity | Transitivity | APL |
| 101 | 208 | 632 | 0.01 | 0.56 | 0.30 | 6.03 |
| 102 | 126 | 219 | 0.01 | 0.52 | 0.34 | 3.45 |
| 103 | 150 | 300 | 0.01 | 0.48 | 0.22 | 6.34 |
| 104 | 291 | 902 | 0.01 | 0.46 | 0.25 | 6.26 |
| 105 | 110 | 255 | 0.02 | 0.51 | 0.25 | 7.28 |
| 106 | 142 | 281 | 0.01 | 0.42 | 0.22 | 6.63 |
| 112 | 276 | 882 | 0.01 | 0.56 | 0.27 | 6.34 |
| 113 | 332 | 1186 | 0.01 | 0.54 | 0.25 | 5.94 |
| 114 | 292 | 931 | 0.01 | 0.50 | 0.24 | 5.89 |

| **Supplemental Table 3. Meta-analysis of changes in friendship networks and e-cigarette use in nine Southern California high schools (N=2,245)** | | | | | | |
| --- | --- | --- | --- | --- | --- | --- |
| **Effect** | **N** | **T^2** | **Mu-hat (s.e.)** | **sigma** | **Q** | **Q p-value** |
| **Network Dynamics** |  |  |  |  |  |  |
| Friend rate (period 1) | 7 | 517.59*** | 16.71 (1.39) | 2.87 | 18.66 | 0.01 |
| Friend rate (period 2) | 9 | 1507.04*** | 6.7 (0.66) | 1.89 | 120.72 | 0.00 |
| Out-degree (density) | 9 | 3282.16*** | -3.41 (0.13) | 0.30 | 26.66 | 0.00 |
| Reciprocity | 9 | 6859.11*** | 2.9 (0.05) | 0.11 | 23.11 | 0.00 |
| GWESP | 9 | 3435.35*** | 1.73 (0.04) | 0.05 | 14.88 | 0.06 |
| Popularity (in-degree square root) | 9 | 81.80*** | 0.19 (0.04) | 0.09 | 22.16 | 0.01 |
| Popularity (Out-degree square root) | 9 | 260.66*** | -0.35 (0.04) | 0.09 | 22.67 | 0.00 |
| Activity (in-degree square root) | 9 | 104.06*** | -0.13 (0.07) | 0.20 | 44.93 | 0.00 |
| Male |  |  |  |  |  |  |
| Alter | 9 | 12.24 | 0.04 (0.02) | 0.00 | 7.05 | 0.53 |
| Ego | 9 | 20.64** | 0.06 (0.03) | 0.06 | 15.34 | 0.05 |
| Homopily | 9 | 694.58*** | 0.5 (0.03) | 0.06 | 19.58 | 0.01 |
| Hispanic |  |  |  |  |  |  |
| Alter | 9 | 20.96** | 0.03 (0.03) | 0.06 | 18.93 | 0.02 |
| Ego | 9 | 22.35** | 0.07 (0.03) | 0.05 | 14.37 | 0.07 |
| Homophily | 9 | 235.80*** | 0.35 (0.06) | 0.14 | 47.59 | 0.00 |
| **Behavior Dynamics** |  |  |  |  |  |  |
| Rate (period 1) | 9 | 12.61 | 0.03 (0.01) | 0.00 | 2.31 | 0.97 |
| Rate (period 2) | 9 | 10.93 | 0.02 (0.01) | 0.00 | 3.88 | 0.87 |
| Rate effects |  |  |  |  |  |  |
| Average exposure | 6 | 12.01* | 3.15 (0.96) | 0.00 | 1.24 | 0.94 |
| Male | 8 | 11.77 | -0.66 (0.23) | 0.00 | 3.67 | 0.82 |
| Hispanic | 8 | 18.95** | 0.76 (0.22) | 0.00 | 6.98 | 0.43 |
| Pro-e-cigarette norms | 9 | 32.42*** | 0.29 (0.06) | 0.00 | 8.79 | 0.36 |
| *p < 0.05; **p < 0.01; ***p < 0.001 | | | | | | |
| N = number of schools on which the statistic for this effect were based; | | | | |  |  |
| Estimate (mu hat) = estimated average effect size; standard error (s.e.); | | | | |  |  |
| sigma = estimated true between-schools standard deviation of the effect size; | | | | | |  |
| T ^2 = statistic for testing that total effect is zero; | | |  |  |  |  |
| Q = statistic for testing that true effect variance is zero, and p-value for associated test | | | | | | |

| **Supplemental Table 4. Logistic regression of individual e-cigarette use on demographic covariates, pro-e-cigarette norms, friend selection, previous network exposure and prior e-cigarette use.** | | | | |
| --- | --- | --- | --- | --- |
|  | **Wave 2**  **(N = 1595)** | | **Wave 3**  **(N = 1909)** | |
| **Effect** | **Estimate (S.E.)** | **AOR** | **Estimate (S.E.)** | **AOR** |
| Male | -0.73 (0.31)* | 0.48 | -0.44 (0.3) | 0.64 |
| Hispanic | 1.3 (0.3)*** | 3.68 | 0.31 (0.29) | 1.37 |
| E-cigarette use at previous wave | 7.58 (1.03)*** | 1954.41 | 8.71 (1.03)*** | 6071.14 |
| Network exposure at previous wave | 1.88 (0.58)** | 6.54 | 1.63 (0.56)** | 5.12 |
| Perceived Pro-e-cigarette norms | 0.27 (0.08)*** | 1.31 | 0.35 (0.08)*** | 1.42 |
| New e-cigarette user friends (selection) | 0.16 (0.15) | 1.18 | 0.48 (0.19)* | 1.62 |
| *p < 0.05; **p < 0.01; ***p < 0.001 | |  |  |  |
